# Establishing a baseline for literature mining human genetic variants and their relationships to disease cohorts

**DOI:** 10.1186/s12911-016-0294-3

**Published:** 2016-07-18

**Authors:** Karin M. Verspoor, Go Eun Heo, Keun Young Kang, Min Song

**Affiliations:** 1 Department of Computing and Information Systems, The University of Melbourne, Melbourne, Australia; 2 Department of Library and Information Science, Yonsei University, Seoul, Korea

**Keywords:** Information extraction, Text mining, Genetic variant information, Biocuration

## Abstract

**Background:**

The Variome corpus, a small collection of published articles about inherited colorectal cancer, includes annotations of 11 entity types and 13 relation types related to the curation of the relationship between genetic variation and disease. Due to the richness of these annotations, the corpus provides a good testbed for evaluation of biomedical literature information extraction systems.

**Methods:**

In this paper, we focus on assessing performance on extracting the relations in the corpus, using gold standard entities as a starting point, to establish a baseline for extraction of relations important for extraction of genetic variant information from the literature. We test the application of the Public Knowledge Discovery Engine for Java (PKDE4J) system, a natural language processing system designed for information extraction of entities and relations in text, on the relation extraction task using this corpus.

**Results:**

For the relations which are attested at least 100 times in the Variome corpus, we realise a performance ranging from 0.78–0.84 Precision-weighted F-score, depending on the relation. We find that the PKDE4J system adapted straightforwardly to the range of relation types represented in the corpus; some extensions to the original methodology were required to adapt to the multi-relational classification context. The results are competitive with state-of-the-art relation extraction performance on more heavily studied corpora, although the analysis shows that the Recall of a co-occurrence baseline outweighs the benefit of improved Precision for many relations, indicating the value of simple semantic constraints on relations.

**Conclusions:**

This work represents the first attempt to apply relation extraction methods to the Variome corpus. The results demonstrate that automated methods have good potential to structure the information expressed in the published literature related to genetic variants, connecting mutations to genes, diseases, and patient cohorts. Further development of such approaches will facilitate more efficient biocuration of genetic variant information into structured databases, leveraging the knowledge embedded in the vast publication literature.

## Introduction

The promise of precision medicine is that individual variation at the genomic level can provide important insights into the detailed disease status of a patient, and guide the selection of the best choice of treatment for that individual. The foundation of this approach is understanding of the relationships between genetic variation, diseases, patient populations, and treatments. Some aspects of this information have been codified into structured biological databases such as the Human Gene Mutation Database (HGMD) [1, 2], COSMIC [3], and the locus-specific gene databases [4]. However, the published literature arguably represents the most up-to-date resource for this information; given the nearly one million biomedical research publications that are indexed in PubMed each year and the challenges facing manual curation at that scale [5].

The Variome corpus [6] is a small corpus of 10 full text journal publications released in 2013 to facilitate the development of text mining systems targeting extraction of information related to the human variome. While small, the corpus has been richly annotated, with 11 entity types including *gene, disease, patient* as well as 13 relation types including *Mutation-RelatedTo-Disease* and *Patient-Has-Mutation*. In total, nearly 7000 entity mentions and over 4600 relation occurrences have been annotated over the corpus. To date, the corpus has been employed to study the performance of mutation extraction systems [7] but has to our knowledge not yet been tested for development of a relation extraction system. In this work, we present a study of the performance of a relation extraction system, PKDE4J, on the Variome corpus. PKDE4J [8] stands for Public Knowledge Discovery Engine for Java; it extracts entity and relations using features derived from a variety of dictionaries and rules, coupled with machine learning to classify relation arguments and semantic type. The system allows for flexible and extensible extraction of entities and relations from unstructured text.

Our study lays the foundation for text mining tools that specifically target the high-value human genetic variation literature. We demonstrate that PKDE4J can be successfully adapted to this task, with an extension to handle multi-relational semantic type classification. We further show that relations can be effectively learned and evaluated using the Variome corpus. However, our results also show that, given good entity recognition, a baseline method of using semantically-constrained co-occurrence is also adequate to achieve comparable performance on the task.

## Background

### Related work

A recent survey of biomedical corpora [9] identifies only a small number of corpora related to genetic variation specifically, including the corpora focused on singular nucleotide polymorphisms (MutationFinder [10], the SNPCorpus [11]), and corpora considering a somewhat broader spectrum of biological sequence information (the OSIRIS corpus [12], and Nagel corpus [13]).

There are even fewer corpora that include relations between the entities related to understanding genetic variation; most studies make use of corpora that are derived through distant supervision from curated resources rather than exhaustive, direct manual annotation of selected articles. A recent study considers the extraction of protein-mutation-disease relations [14], achieving a reported F-score of 0.643 on this task, and is based on a benchmark data set of PubMed abstracts derived using the curated relations in SwissProt.

We acknowledge the recent release of two corpora that were not yet available at the time that we initiated our study; the BRONCO corpus [15] and the BiomutaC data developed for the DiMeX system [16]. The Variome corpus is differentiated from these corpora in that it specifically includes relations connecting patient cohorts to mutations and diseases, allowing for detailed extraction of the characteristics of specific subgroups of patients described in the literature. Both of these new corpora focus on extracted associations rather than text-bound relations; they do not adopt a standard representation for corpus annotations (e.g., BioC [17] or brat [18] format) and hence it is difficult to identify specific annotations of relations that are tied to specific text spans. While the extracted associations (e.g., the <Mutation, Gene, Disease > triples that are the target of DiMeX) are provided, it is not made explicit where in the text these relations are expressed in either corpus. We further note that the BRONCO corpus has not been used to test relation extraction methods.

A number of corpora are available for the broader task of biological information extraction, but do not specifically consider genetic variants and their relations. The CoMAGC corpus [19] does include manually annotated relations, but focuses on gene-cancer relations, and specifically addresses the role of a gene in a given cancer; it is focused on the underlying biology rather than the impact of genetic variation and therefore does not include mutation information. AIMed [20] contains 200 abstracts with the annotation of protein-protein interactions. BioInfer [21] is a corpus that contains 800 articles annotated with genes, DNA, proteins and so on. The HPRD50 [22] corpus, used to develop the rule based relation extraction system RelEx, contains 50 abstracts and annotations about protein-protein interactions. The Craven [23] corpus consists of binary relation annotations; protein-protein interaction and gene-disease association. The EDGAR [24] corpus is annotated with binary relations between gene, drugs, and cells.

There exist a few systems that are aimed extracting the same relations that we are interested in from the literature and make use primarily of co-occurrence strategies. These include PolySearch [25] and SNPShot [26]. PolySearch includes diseases, genes, mutations, and drugs, but was only assessed on protein interaction and disease gene relations. SNPShot was assessed primarily with respect to the PharmGKB database, but included a small manual assessment of a set of relations including gene-disease, gene-variant, and gene-drug relations, as well as a very small number of variant-population examples; overall F-score in that evaluation ranged from 0.62–0.84. While the Variome corpus is small, it contains full text articles rather than abstracts, and therefore has substantially more examples of these relation types.

## Methods

In this section, we present the models we developed. We combine the various approaches adopted in prior literature to build a model that can be used for relation trigger and entity extraction suited to biological relation extraction.

We demonstrate this by testing our models on the Variome corpus, the results of which are presented in the next section.

**Fig. 1:**
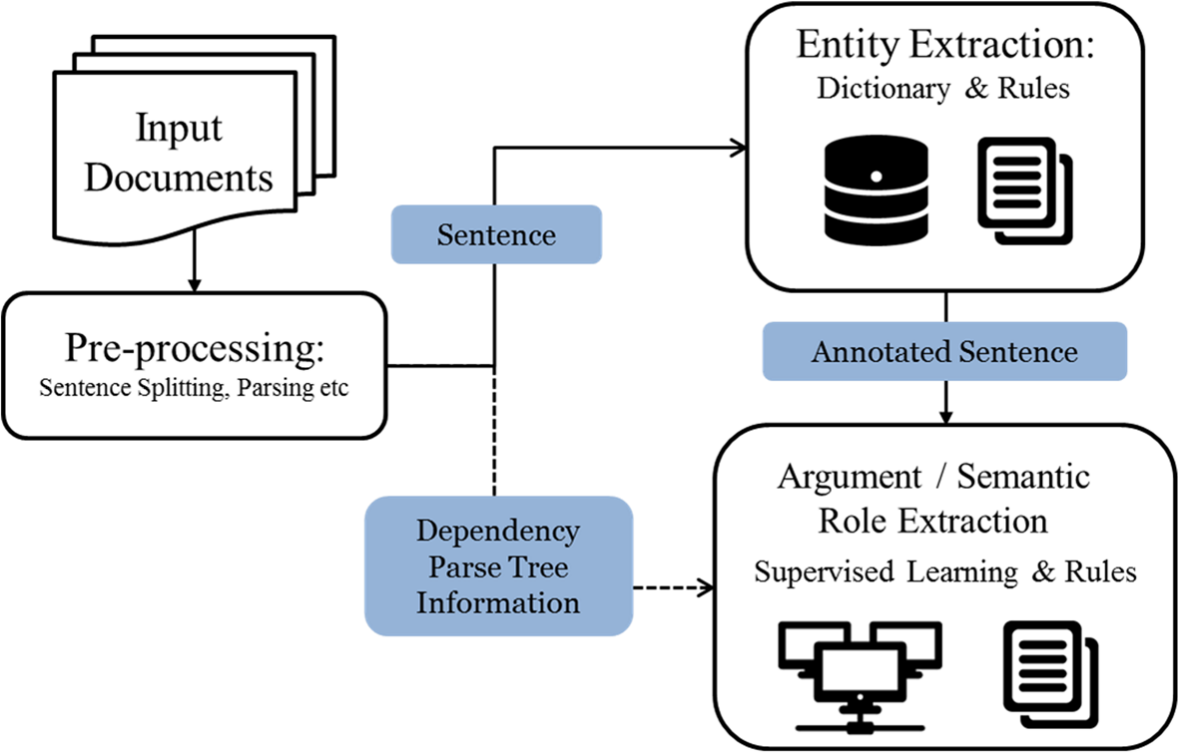
Overall architecture of the proposed approach

**Fig. 2:**
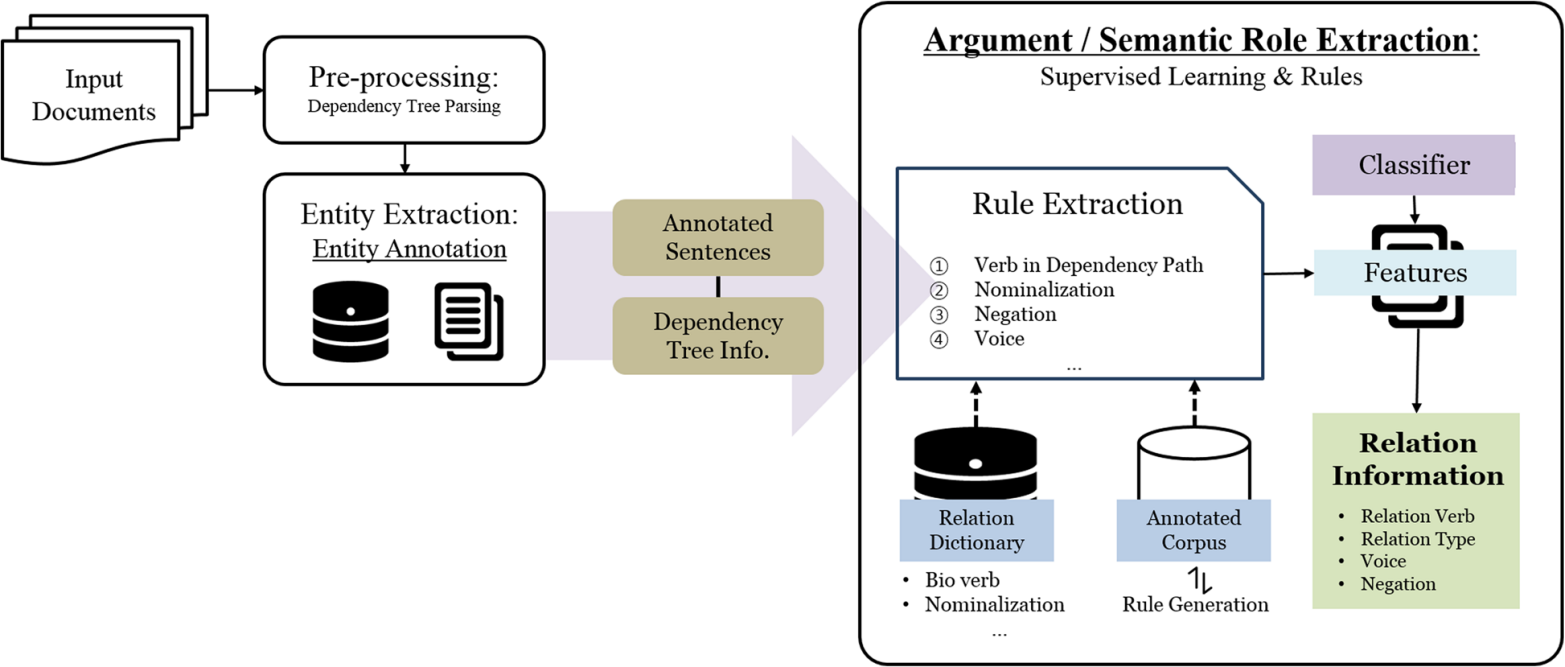
Architecture of PKDE4J. The structure of the overall PKDE4J system

In this paper, we extend PKDE4J [8], which stands for Public Knowledge Discovery and Extraction for Java (Fig. 1). PKDE4J is a flexible and extensible entity and relation extraction system based on dictionaries and rules. The user can modify the dictionaries and rules for extracting specific entity and relation targets. The overall architecture of PKDE4J is shown in Fig. 2. The performance of this system achieves an average F-measure 85 % for entity extraction and 82 % for relation extraction across a number of tested corpora [8]. This flexible text-mining system is publicly available at http://informatics.yonsei.ac.kr/pkde4j. PKDE4J adopts the system architecture of Stanford CoreNLP, with the annotation pipeline consisting of tokenization, lemmatization, dependency and constituency parsers, POS taggers, etc. The entities and their relationships are represented as annotations on the already existing sentence annotations, by implementing the CoreAnnotation interface that is provided in the CoreNLP tool.

Relation extraction for the Variome Corpus using PKDE4J includes the following steps:

1. Determine features for relation extraction.
2. Identify the entities that are arguments of a relation.
3. Assign the semantic type of relation to the argument-entity pairs identified.

We describe each of these steps in a subsection below.

### Feature determination

We model relations and entities as sub-graphs headed by a node in the dependency tree. The basic assumption is that each sentence is independent of each other from the perspective of entity-entity relationships, i.e. that a relation is expressed within the boundary of a sentence. Relations are denoted by specific words that introduce the relationship (their trigger words) and hence are pre-terminals in the parse tree.

Entities are denoted by a parse tree-node covering a sub-tree that spans over the whole text of the entity. In some cases, there is no single node that covers the entire entity (mostly because of parser errors, for e.g., PP attachment). In this case, we use an approximation to mark entities, by first repeatedly removing tokens from the end of the span to find a single subtree that covers the full text. Otherwise, we repeat the same process from the beginning of the span of text. We manually verified that this heuristic works well in practice and results in entities that convey almost the full meaning of original span, and are well-formed.

For feature generation, we make use of the dependency graph of the sentence. The features are extracted by identifying the position of the headword of an entity or relation trigger from the dependency tree and analyzing the properties of the head word. We use the Collins head-finding algorithm to find the head word of a parse tree node [27].

Each feature is primarily based on how two entities are related in a sentence, using either syntactic or lexical properties of tokens. Using PKDE4J, a relation is established when a pair of biological entities is linked by a directed arc. The relation is usually labeled as having a semantic type that the relation has (e.g., *RelatedTo* or *Has*). When determining the relation type, we further identify information helpful for the relation detection such as negation and voice. The result of syntactic dependency parsing is dependency relations among tokens (with a syntactic dependency as predicate, and tokens as arguments). This is useful information for detecting the semantic relations expressed in the sentence.

PKDE4J includes a set of 19 rules that capture syntactic and lexical characteristics of sentences, which in turn are used as features for relation extraction. Extending the 17 rules described in the PKDE4J paper [8], two lexical features were added. These were *window left entity* and *window right entity*, in order to represent the words in the context of a particular entity. Also, the rules were organized in terms of the categories of syntactic and lexical features. But, these changes reflect general improvements and were not specifically revised for the Variome corpus.

These rules were developed through manual analysis of 1,000 randomly sampled sentences from PubMed.

A dependency parser was applied to these sentences; the parses were analyzed, and general rules were systematically proposed based on general characteristics or structures that are common across sentences. These in turn can be applied for spotting possible relations in other sentences. To validate each rule, we tested it on the same corpus repeatedly. Through this analysis and validation process, finally the 19 rules are formalized. These rules are combined in various ways, and considered in different orders, depending on the target relation extraction task/corpus. Not all rules are necessary for each task. New rules can also be added, depending on the traits of each task. The strength of PKDE4J is in this point; the system is flexible and extensible to be applied to other corpus or tasks.

#### Syntactic features

We extract syntactic features for rule generation. To generate these features we parse the sentence using Stanford dependency parser. Table 1 summarizes the rules for syntactic feature generation.

**Table 1:** Rules for syntactic feature generation

Rule name	Description
Verb in dependency path	In the level of root (verb), shows subordinate dependency relation types and directions
No verb in dependency path	Determine whether the sentence has a verb or not between two entities. If not, detect nominalization or weak nominalization rule is processed
Contains clause	Check if the sentence has any clauses
Clause distance	Distance between clause and entities in left and right, the closest ones. The entities might be all to the right or to the left or divided
Same head	Check if two entities have same parent
Full tree path	Use it in dependency parsing process
Path length	Path length from the parent node to child node

**Verb in Dependency Path** When a verb occurs in the dependency path between two biological entities, the dependency relations involving the verb, including the directionality of the dependency relations (such as *subj → prep_of ← mod*), are selected by this rule. The verb has a central role in this rule, because it may be a trigger word of a biological relation. Either left, right, or both directions of the path anchored on the verb can exist. By applying this rule, we extract a concatenated set of relations between the two entities through the dependency tree.

Example: (*Sentence*) “EPA reduce the vasoconstriction in a dose-dependent manner”; (*Output*) EPA [GE] → reduce (POS_VB): nsubj AND reduce (POS_VB) ← vasoconstriction [BP]: dobj → Relation Verb = DOWN_REGULATE.

In the above example, there are two biological entities, EPA (gene) and vasoconstriction (biological process). The verb “reduce” is linked to these entities in different directions of the dependency tree. Thus, verb in dependency path rule detects the term “reduce” as a verb of the relation existing between the two entities.

**Contains Clause** In a sentence, the existence of a dependent clause is an important feature for relation extraction. Through the clause determination process, we can identify any clause embedded in the sentence, and recognize the the start and end tokens of the clause. We also consider the chunk tag of *SBAR* from Penn Treebank [28], which denotes clause by a subordinating conjunction. If the *SBAR* is located between the two entities, then each occurs in a different clause and there is unlikely to be a relation between them.

#### Lexical features

The lexical features describe specific words between and surrounding the two entities in the sentence in which they appear, or capture lexical characteristics of the sentence. Each lexical feature consists of a vector of all words identified by the rule for each entity *e* and appended, or based on the entity pair together (depending on the nature of the rule). Each row in Table 2 represents a single lexical feature that is considered. Part-of-speech tags were assigned by a maximum entropy tagger trained on the Penn Treebank, and then simplified into seven categories: nouns, verbs, adverbs, adjectives, numbers, foreign words, and everything else.

**Table 2:** Rules for lexical feature generation

Rule name	Description
Negation	Determine negation by checking existence of negative word e.g. a negative adjective modifying the verb or relation trigger, or a semantically negative word
Tense (active/passive)	Tense determination
Words in between entities	The sequence of words between the two entities
Surface distance	Distance between the two recognized entities (including existing tokens and entity itself)
Window left entity	A window of *k* words to the left of Entity 1, with their part-of-speech tags
Window right entity	A window of *k* words to the right of Entity 2, with their part-of-speech tags
Detect nominalization	Existence of nominalized verb located in left/right position of the entity and distance from specific entity
Weak nominalization	Detect when an entity occurs after a preposition and whether a nominalized biomedical verb is located ahead of that preposition

**Negation** In PKDE4J, the judgment of negation does not capture general sentence negation, but narrow the scope to negation impacting the relation between the two relevant entities. We utilize the dependency relation *NEG*, which represents a relation between the negation word and the modified word.

**Nominalization** Nominalized forms of words are prevalent in the molecular biology sublanguage to represent complicated interactions of molecular entities. This form is more difficult to detect than verbs [29]. Because of the limited solution for detecting nominalization, we manipulate the extant alternations related to the argument structure of nominalizations using the dependency tree structure. The template for nominalization is as follows:



**Weak Nominalization** In the situation where the nominalized form of words are not detected because of the strict rule set, we add the rule for the loosely defined pattern of nominalization when only one entity satisfies the pattern of nominalization.

#### Supplementary features

In addition to those core relation rules described above, we provide a set of supplementary rules such as Number of entities between two entities, Surface distance, Entity counts, Same heads, etc. (Table 3). One of the important supplementary features is based on named entities. Every feature contains, in addition to the content described above, named entity tags for the two entities. We typically perform named entity tagging using PKDE4J, although for the experiments described below we work with provided entity tags.

**Table 3:** NER-based Rules for supplementary feature generation

Rule name	Description
Number entities between	Number of recognizable entities located
entities	between the two recognized entities
Entities in between	Show which entities are located in between
	the two main entities
Entity counts	Number of entities
Entity order	Order of entities

**Voice** The voice is utilized in determining the passive auxiliary by using dependency relation auxpass. A passive auxiliary in clause is not a main verb but indicates passive meaning. (e.g. *TP53 is regulated*; auxpass (regulated, is)).

### Argument identification

We apply a maximum entropy model to identify entities that are arguments to relation triggers. We represent the entity extracted from the full-text as a node in a parse tree. Since multiple relations may exist in a sentence, we need to predict whether and how a node is bound to a trigger word. To this end, we compute a probability for each node and determine whether the node is an entity associated with each relation. Given a relation trigger *e* and *M*, the set of *m* nodes {*i*_1_,*i*_2_,…*i*_*n*_} in the parse tree, we are to predict the best labeling, *A* of nodes to either *ARG* or *NONE* for the relation trigger *e*.

Identification of relation trigger words can vary in difficulty depending on the relation type. For protein-protein interaction (PPI) relations, only a limited number of trigger words are relevant and a sophisticated dictionary is enough for identifying them. But for other relation types, a simple dictionary based approach is not sufficient. Thus, we adopt a binary classification model of MaxEnt to classify a node as either an *ARG* (argument) or *NONE* (not an argument), using a feature representation using the features outlined in the previous section.

**Fig. 3:**
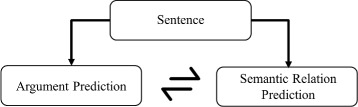
Stages in iterative optimization algorithm

For our classification tasks, we use maximum entropy (MaxEnt) models based on an implementation of L-BFGS that stands for the Limited-memory Broyden-Fletcher-Goldfarb-Shanno algorithm [30]. It is an optimization algorithm in the family of quasi-Newton methods for parameter estimation, and the general description of the algorithm is founded in Nocedal and Wright’s book [31]. Since the information about entities can be used to improve relation trigger prediction, we use an iterative optimization algorithm to iteratively predict relation triggers from entities and entities from relation triggers (Fig. 3).

In prediction of a node as an argument to an relation trigger, predicted entities are often duplicates across the sentences. To tackle this issue, we adopt the dynamic programming approach proposed by Toutanova et al. [32] by utilizing the probability of the node and its immediate children being entities. Starting from the pre-terminal nodes of the tree, the algorithm attempts to find best assignments for each sub-tree using the already computed assignments for its children to make sure that a sub-tree that is a part of *A* in itself doesn’t have smaller subtrees that belongs to *A*.

### Semantic relation

For argument identification, we use a binary classifier based on the MaxEnt model that predicts whether an entity is an argument to a trigger. For semantic relations, we extended this model to a multi-class classifier to generate probability values for a parse-tree node being assigned to a specific semantic relation type. We also modified the dynamic program for non-overlapping constraint used in argument prediction to a re-ranking model that jointly assigns semantic role to all nodes in a sub-tree. Let *L* be a labeling of semantic roles to the parse tree nodes, including *NONE* if it is not an entity. Our goal is to maximize the probability of the labeling we generate.

We use a bottom up re-ranking approach by keeping the top-*k* joint assignment of semantic relation types to all nodes in a sub-tree. This algorithm is similar to the dynamic programming for non-overlapping constraints. At the pre-terminal nodes, we keep just the semantic relation types of the word subsumed by the node in descending order of probability. At nodes above the pre-terminal nodes, there are two scenarios:

1. The node is an argument to the trigger: In this case, the node has a non-*NONE* semantic relation type and none of the children nodes can be entities. Hence all children would have semantic relation type *NONE*. For each non-*NONE* semantic relation type of the node, we compute the probability value of the joint labeling of the sub-tree.
2. The node is not an argument to the trigger: In this case, the node has a semantic relation type of *NONE*. Since at each of the child nodes we have top-*k* possible assignments, we take all combinations of assignments of children nodes and compute the probability for each of these possible joint relation.

### Experimental set-up

#### Studied Variome relations

**Fig. 4:**
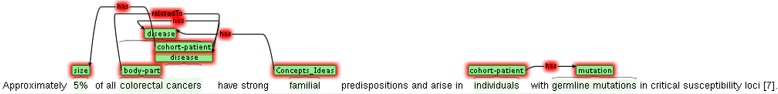
Example Variome annotation. An example sentence annotation from the Variome corpus [6], including annotations of a number of relations such as *cohort-has-mutation* (*“individuals with germline mutations”*) and *cohort-has-size* (“*5 % of all colorectal cancers”*, where ‘cancers’ is treated as a metonym for a patient cohort)

For the purposes of our study, we selected relations from the Variome corpus annotations that have at least 100 examples. An example of a single sentence annotated with a number of different entities and relations in the corpus is shown in Fig. 4. The most frequent relations in the dataset are *cohort-has-disease*, indicating the disease that the study group is associated with, and *cohort-has-size*, capturing the number of patients in the study group. Other well-represented relations include *gene-has-mutation*, which specifically identifies in which gene a given mutation occurs, and *cohort-has-mutation*, indicating an association between a study group and a genetic variant.

The original Variome Annotation Schema included a single category of *characteristic* that was fairly vaguely defined as “a characteristic of disease or tumour, in the sense of a property or feature that commonly occurs in or is associated with that disease or tumour” [6]. However, the inter-annotator agreement for this category proved low, and it was divided into subtypes based on mappings to UMLS Semantic Groups [33]; specifically *Concepts & Ideas*, *Disorders*, *Physiology*, or *Phenomena*. We therefore primarily focused on relations involving this finer-grained breakdown of the relations involving *Characteristics*, and the relations involving other entity types. With our 100-example threshold, this left us with 11 relations, involving *gene*, *disease*, *mutation*, *cohort/patient* (hereafter referred to simply as *cohort*), and *body part* entities, plus the subtypes of *characteristic*.

Splitting *Characteristics* into these subtypes impacts the relation extraction system in two ways; first, by splitting relations such as *Disease-has-Characteristics* into four (one for each subtype) and second, by enabling inference of more targeted patterns targeted to the usage contexts of each different subtype. The first issue means that each relation in the split scenario has a smaller number of examples, potentially impacting the ability of the system to learn patterns that generalize to new contexts, limiting recall, while the second means that the split scenario is likely to result in higher-precision patterns. We therefore also ran an experiment with the four *Characteristics* subtypes collapsed back into a single category, for comparison purposes.

#### Evaluation

We treat each relation as a binary entity-relation tuple. Given a relation *r*∈*R* and a tuple *t*∈*T* the pair <*r,t*> is a relation instance. That is, each relation is defined in terms of core relation that connects them (*has*, *relatedTo*) plus the types of the arguments of the relation. As such, *Gene-has-Mutation* is treated as a distinct relation from *Cohort-has-Mutation* and *Cohort-has-Disease*.

We focus on relation extraction in our evaluation. Therefore, we use as a starting point for training the system the entity annotations in the gold standard. That is, the entity annotations are taken as given (provided), rather than needing to be inferred automatically. This gives us a ceiling for performance of the end-to-end pipeline in a real-world setting, by removing any potential errors in the entity annotation from the evaluation results. Some of the relations that we work with involve entity categories that may be difficult to identify (specifically, the *Characteristics* category), and some entity types are not exhaustively annotated but only have relevance for a given set of relations (specifically, the *Size* category). We avoid these complexities by starting both the training and assessment of the relation extraction system with the gold entities provided.

**PKDE4J learning set-up** We trained a classifier for each selected relation individually. We employed 10-fold cross-validation, and measured the results in terms of the standard metrics of Precision, Recall, and F-score. We randomly permute the order of files to avoid similarities in adjacent files and then use 10 fold cross validation on the training set.

**Baseline co-occurrence system** We implemented a simple sentential co-occurrence baseline to measure the contribution of the PKDE4J system on this task. In this set-up, we start with the annotated entities from the gold standard, and extract any pairs of entities within a sentence boundary that satisfy the semantic constraints of a relation (e.g., matching the argument entity types for the <*r,t*> tuple) as an instance of that relation. This is the simplest possible relation extraction system. Co-occurrence based methods for relation extraction have shown value in other work, especially in a predictive context [34, 35].

For example, for the sentence in Fig. 4, we would extract the *Cohort* annotations of *cancers* and *individuals* and the *Disease* annotations of both *colorectal cancers* and *cancers*, and infer a *Cohort-has-Disease* relation between each of the combinations of these, e.g., *individuals-has-cancers*, *cancers-has-cancers*, *individuals-has-colorectal cancers*, and *cancers-has-cancers*.

We expect a co-occurrence system to have high recall as compared to any other system; in principle it will identify all valid relations in a given sentence. However, the Variome corpus includes relations that are not sentence-bound, and therefore there is the possibility of less than perfect recall.

## Results and discussion

The results are presented in Table 4, using a Precision-weighted F- *β* score calculation with *β*=0.5. The baseline co-occurrence method sets an upper bound for the Recall of any sentence-bound method; as expected, the Recall of PKDE4J does not exceed the baseline Recall for any relation type given the assumption made by PKDE4J that all relations between entities occur within a single sentence, and that it starts with two entities in a sentence. PKDE4J can only improve the Precision over the baseline method; it will effectively work to filter out co-occurrences that do not correspond to a meaningful relation.

**Table 4:** Relation extraction results over Variome corpus relations with at least 100 examples, based on 10-fold cross-validation and assuming gold-standard entity annotations

Relation	N	Baseline			PKDE4J		
		P	R	F	P	R	F
Disease-has-ConceptIdeas	431	0.704	0.922	0.764	0.799	0.746	**0.781**
Disease-has-Physiology	188	0.752	0.873	0.788	0.885	0.684	**0.806**
Disease-has-Disorders	349	0.754	0.884	**0.793**	0.773	0.713	0.752
Disease-relatedTo-BodyPart	445	0.763	0.853	**0.791**	0.794	0.666	0.746
Mutation-relatedTo-Disease	126	0.702	0.986	**0.777**	0.683	0.973	0.758
Gene-has-Physiology	180	0.844	0.897	**0.861**	0.857	0.723	0.807
Gene-has-Mutation	538	0.835	0.866	**0.845**	0.910	0.569	0.758
Cohort-has-Mutation	307	0.873	0.921	**0.888**	0.909	0.726	0.839
Cohort-has-Disease	717	0.715	0.813	0.745	0.865	0.654	**0.781**
Cohort-has-Size	669	0.857	0.793	0.835	0.910	0.736	**0.844**
Cohort-has-Disorders	119	0.859	0.918	**0.878**	0.903	0.748	0.845

P = Precision, R = Recall, F = F0.5 score (weighting P more than R due to the importance of Precision). The {P,R}-Base results refer to results from a simple co-occurrence baseline. The best F score (F-Base or F-PKDE4J) is bolded

Indeed results of PKDE4J on the Variome corpus as compared with the baseline co-occurrence method demonstrate that there is consistent value in applying the PKDE4J method to increase Precision. PKDE4J outperforms the baseline method in Precision nearly across the board (in all but two cases; for *disease-has-conceptIdeas* this difference is only 0.005 while for *mutation-relatedto-disease* it is 0.02).

Overall, the two systems achieve comparable performance as measured by F-0.5 score, showing that the increased Recall of the baseline system often outweighs the impact of the improved Precision of PKDE4J, despite emphasizing Precision in the F score calculation. This indicates that the use of gold entities, plus strict application of semantic constraints on the arguments of specific relation types, is adequate in the vast majority of cases to identify a valid relation within a given sentence. This result is supported by prior research demonstrating the value of semantic constraints on relation extraction [36].

The results for both methods are also higher than the reported results from SNPShot [26] on their data set (which has not been made available), indicating that the annotated relations in the Variome corpus are valuable as training data. More analysis is needed to understand the differences in the tasks addressed in these approaches, but at a methodological level the primary difference is that PKDE4J makes the most use of linguistically-based relation extraction strategies.

Overall, the performance of PKDE4J is good, with no relation resulting in less than 0.74 F-score. Given the inter-annotator agreement results over the doubly-annotated portion of the Variome corpus as reported in [6], which ranged from 0.62–0.90 (prior to consensus-based re-annotation) for the most frequently attested relations, the results of PKDE4J are close to human performance on this task.

For this initial experiment, we did not control the baseline co-occurrence system for overlapping annotations that could be automatically filtered. In general it probably does not make sense to have a given entity related to another possible entity interpretation of the identical string. For instance, for the sentence in Fig. 4, the *Cohort-has-Disease* relation *cancers-has-cancers* is based on annotation of the same underlying term in the sentence (“*cancers*”) in two distinct ways. It would not be common to relate one interpretation of a term to another interpretation of the same term. In future analyses, we will filter such relations out; coupled with more sophisticated strategies to improve co-occurrence methods such as employed in the SNPShot system [26], the co-occurrence approach could provide a stronger baseline.

The results of our experiments with the collapsed *Characteristics* category appear in Table 5. Compared to the results in Table 4, we see generally lower performance, indicating that the potential benefit to generalization from the increased number of training examples is outweighed by a lack of common patterns between those examples. This supports the use of the finer-grained relations in terms of *Characteristics* subtypes, as proposed in the analysis of [6].

**Table 5:** Relation extraction results over Variome corpus relations, with the *Characteristics* entity type aggregating its subtypes (*Concept & Ideas*, *Disorders*, *Physiology*, and *Phenomena*) based on 10-fold cross-validation and assuming gold-standard entity annotations

Relation	N	P	R	F
Disease-has-Characteristics	989	0.749	0.602	0.667
Mutation-has-Characteristics	17	0.701	0.583	0.642
Gene-has-Characteristics	230	0.876	0.699	0.778
Cohort-has-Characteristics	239	0.886	0.734	0.803

P = Precision, R = Recall, F = F1 score

## Conclusion

We have provided the first results for automatic relation extraction on the range of complex relations captured in the full-text Variome corpus, and extended the PKDE4J system to suit relation extraction at a fine-grained level over a number of different relation types. The Variome corpus captures the core information relevant to genetic variant databases. The system results are promising, outperforming a co-occurrence baseline on Precision in most cases and approaching human-level agreement on relation extraction. The co-occurrence baseline sets a strong benchmark, due to the strong semantic constraints on the relation definitions (e.g., a *cohort-has-disease* relation must involve a *cohort* entity and a *disease* entity); it appears that few sentences in the Variome corpus involve mentions of multiple cohorts or multiple diseases, where relevant entity pairs are unrelated.

In future work, we will analyze these results in more detail, in order to identify specific challenges in the extraction of relations connecting genes, diseases, patients, and mutations and identify strategies for further improvements, and to further explore the impact of simple semantic constraints on relation extraction. The current results are sufficient to give us confidence that an automated approach to extraction of this valuable information is feasible. We therefore plan to use this system as a foundation for large scale information extraction of genetic variant relationships from the published literature, to augment existing manual efforts.

We have identified some avenues for future improvements to the application of PKDE4J to this task on the basis of this work. The current application of the method to the Variome corpus treats relation trigger and argument prediction independently, since entities pre-identified in the gold standard were used. Therefore, an error in trigger identification has a direct impact on argument identification. To resolve this problem, we plan to make use of the entities manually identified in the training data to enrich the feature set for relation trigger prediction. This is based on the assumption that more entity information enhances relation trigger prediction. In our case, as the entities manually identified in the training data are unavailable during the test phase, we will implement an entity prediction model which is independent of the triggers. We plan to explore the procedure that is mentioned in the argument identification section for this purpose. We will also explore the integration of existing named entity recognition tools for the relevant entity types (mutations, genes, diseases, ethnicity, etc.) to enable measurement of an end-to-end solution to this information extraction task.
